# TRPM7 in Peripheral and Central Immune Cells: Emerging Roles in Neuroinflammation and Neurological Disease

**DOI:** 10.3390/ijms27125157

**Published:** 2026-06-06

**Authors:** Kyra S. Brewer, Zhi-Gang Xiong, Tiandong Leng

**Affiliations:** Department of Neurobiology, Morehouse School of Medicine, Atlanta, GA 30310, USA; kbrewer@msm.edu (K.S.B.); zxiong@msm.edu (Z.-G.X.)

**Keywords:** TRPM7, TRP, ion channel, immune cells, neurological diseases

## Abstract

Transient receptor potential cation channel, subfamily M, member 7 (TRPM7) is a unique protein that functions as both a nonselective cation channel and an alpha kinase (chanzyme). It is ubiquitously expressed across a wide range of tissues and cell types. Through its chanzyme activities, TRPM7 is implicated in many fundamental processes such as intracellular cation homeostasis, cell growth, proliferation, differentiation, and cell cycle progression. Increasing evidence has revealed a crucial role of TRPM7 in regulating immune cell development, activation, and inflammatory responses. This review summarizes recent advances in understanding TRPM7’s structure, function, pharmacology, and roles in innate and adaptive immune cells. In particular, we discuss its impact on immune cells in the central nervous system and its potential implications for neuroinflammatory and neurodegenerative diseases.

## 1. Introduction

Inflammation represents a coordinated biological response to infection, tissue damage, and cellular stress, involving interactions among diverse immune cell populations and signaling pathways. While acute inflammation is essential for host defense and tissue repair, chronic or dysregulated inflammation contributes to the pathogenesis of a wide range of diseases, including autoimmune disorders, cardiovascular disease, metabolic syndrome, and neurodegenerative diseases [1]. Elucidating the regulatory mechanisms governing immune cell activation and downstream inflammatory signaling is, therefore, critical for developing effective therapeutic interventions.

TRPM7 (also termed TRP-PLIK, ChaK1, or LTRPC7) is a member of the TRPM subfamily within the larger TRP ion channel superfamily [2]. It is a bifunctional “chanzyme” that combines ion channel activity with a C-terminal α-kinase domain and is one of only three such proteins identified among mammalian ion channels [3]. TRPM7 is broadly expressed across all tissues, with the highest levels reported in the heart, pituitary gland, bone, and adipose tissue [3,4]. In mice, TRPM7 is present in almost all tissues and has a strong expression in the brain [5,6]. The TRPM7 channel is a master regulator of intracellular divalent cations, particularly Mg^2+^, thereby influencing a wide range of physiological processes, including enzymatic activity, cell survival, and early embryonic development [7]. In parallel, TRPM7-kinase possesses the intrinsic capability to phosphorylate several downstream protein substrates, modulating diverse signaling pathways that govern cell proliferation, migration, and stress responses [8,9,10]. TRPM7 emerges as a key player in cardiovascular diseases [11,12], and various forms of cancer [13,14,15,16]. For example, TRPM7 enhances the malignant phenotype of glioblastoma, promoting proliferation, invasion, and migration [15,16]. In addition, over-activation of TRPM7 has been shown to exacerbate neuronal injury by inducing Ca^2+^ and Zn^2+^ overload [17,18].

Accumulating evidence supports that TRPM7 may also function as a critical regulator of immune function in both innate and adaptive immune cells, including macrophages, dendritic cells, neutrophils, T cells, and B cells [19,20]. Through its dual channel and kinase activities, TRPM7 orchestrates key processes such as immune cell activation, cytokine production, migration, and differentiation [21,22,23,24]. This body of evidence is derived predominantly from peripheral immune cells, and the role of TRPM7 in CNS-resident immune cells, particularly microglia, remains poorly defined. Given its established immunomodulatory functions in peripheral immune cells, TRPM7 is likely to regulate microglial activation and function, thereby contributing to the progression of CNS diseases, e.g., stroke and multiple sclerosis, which are characterized by pronounced neuroinflammation and neuronal cell death [25,26]. This review will summarize the current knowledge of TRPM7 in peripheral immune cell functions and discuss its emerging role in CNS immune cells, particularly microglia and astrocytes, as well as its potential contributions to neuroinflammatory and neurodegenerative diseases.

## 2. TRPM7 Structure and Function

### 2.1. TRPM7 Structure

**Figure 1:**
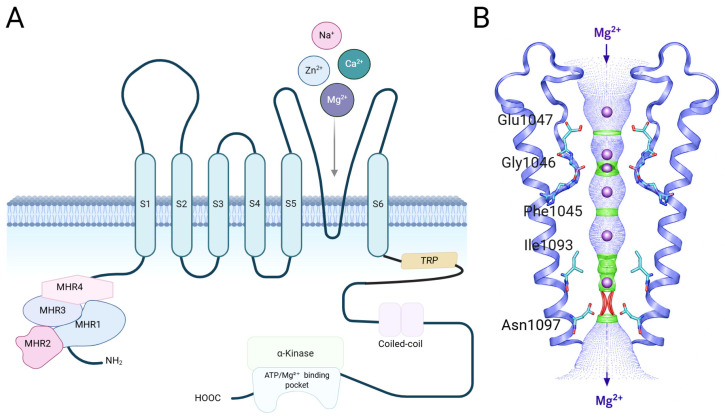
Structure of TRPM7. (**A**) TRPM7 forms a tetramer, and each subunit is made up of an N-terminal cytosolic region containing melastatin homology regions (MHR1–4), six transmembrane domains (S1-S6), a TRP domain, a coiled-coil domain, and a C-terminal domain. MHR Domain: This is unique to TRPM channels. The transmembrane domains include the voltage-sensor-like domain (S1–S4) and channel conducting pore loop (S5–S6). The channel pore conducts Mg^2+^, Ca^2+^, Zn^2+^, and Na^+^. TRP domain: This is a short helix just after S6 that stabilizes the channel and links to gating mechanisms. Coiled-Coil domain: This is immediately after the TRP domain in the cytoplasm. Acts like a “hub” to hold the four subunits together. It helps in channel gating and kinase–channel communication. α-Kinase Domain: This is an α-kinase (recognizes α-helical substrates) instead of a classical serine/threonine kinase fold. It phosphorylates intracellular proteins (including myosin IIA heavy chain) to regulate the cytoskeleton, migration, and proliferation. ATP-binding pocket: In crystal structures, it resembles other α-kinases but has TRPM7-specific loops for channel–enzyme coupling; the linker between channel and kinase transduces conformational changes from the channel opening to modulate kinase activity, and vice versa. It also senses metabolic state (e.g., Mg^2+^/ATP levels) to tune channel function. (**B**) Ion conduction pathway. The funnel-shaped selectivity filter is formed by Glu1047, Gly1046, and Phe1045 in the pore helix. Asn1097 forms narrow constriction sites at the lower gate.

Like other TRP channels, TRPM7 is a tetramer assembled from four identical subunits [27]. Each subunit has six transmembrane-spanning domains (S1–S6), with a pore-forming loop between S5 and S6, and both C and N termini are located intracellularly (Figure 1A). The N-terminal cytosolic region, comprising approximately 600–700 amino acids, contains four melastatin homology regions (MHR1–4) that are linked to the transmembrane-spanning domain [28]. S1–S4 is the voltage-sensor-like domain, which is not as strongly voltage-sensitive as classic voltage-gated channels [27]. The selectivity filter of TRPM7 is formed by the backbone carbonyls on the side chains of Phe1045/Gly1046 and Glu1047 [27] (Figure 1B). The lower gate of the channel contains two constriction sites (Ile1093 and Asn1097), with the most constricted point at Asn1097, where S6 helices from all four subunits form a hydrophobic seal. The disulfide bond in the pore loop between Cys1056 and Cys1066 is essential for divalent cation permeability, a common feature of the TRPM family [27]. A Cryo-EM reconstruction density map of TRPM7-Mg^2+^ suggests that Mg^2+^ ion is located at the most restricted site of the selectivity filter in the ion permeation pathway [27].

The transmembrane-spanning domain ends with the amphiphilic TRP domain (TRP Loop) (Figure 1A), which runs almost parallel to the surface of the membrane near the S6 helix [27]. This domain is a signature and highly conserved within the TRP channel family. It is proposed to participate in subunit assembly or allosteric modulation of channel gating [29,30]. Upon TRPM7 channel opening, TRP helices become shorter, while the S6 helices extend, which causes the movement of S5 away from the pore center and substantial dilation of the channel pore [27]. The TRP domain is followed by a cytoplasmic coiled-coil (CC) domain, which is proposed to participate in tetrameric chanzyme assembly by bundling the membrane-proximal C-terminal [31]. The crystal structure reveals that the kinase domain of TRPM7 bears a striking resemblance to that of classical protein kinases, but there are significant differences in its C-terminal lobe, which resembles ATP-grasp fold enzymes [32]. The C-terminal lobe contains a metal ion, zinc, which is integrated into the hydrophobic core of the C-terminal lobe and is expected to be crucial for the stability of the kinase domain [32]. Disruption of the zinc-binding module results in the complete loss of kinase activity [32].

### 2.2. TRPM7 Channel Function

The TRPM7 channel exhibits a unitary conductance of approximately 40 pS. Under whole-cell voltage-clamp conditions, TRPM7 currents show a characteristic outward rectification, with a reversal potential close to 0 mV [33,34]. Unlike most other TRP channels, TRPM7 is permeable to divalent cations including Ca^2+^, Mg^2+^, and Zn^2+^, in addition to monovalent cations such as Na^+^ and K^+^ [33]. The channel displays constitutive activity and is tightly regulated by Mg^2+^, ATP, pH, and PIP_2_. The inactivation/deactivation of TRPM7 is enhanced by PIP2 hydrolysis, intracellular Mg^2+^, and intracellular acidification [28,35,36]. In contrast, removal of external divalent cations, extracellular acidosis, or addition of glutathione can potentiate TRPM7 currents by increasing their permeability to monovalent cations [28,37,38]. Under physiological concentrations of Mg^2+^, TRPM7 currents are typically small; however, when extracellular Mg^2+^ is reduced, and cells are dialyzed with Mg^2^-free intracellular solution during whole-cell patch-clamp recordings, large outwardly rectifying currents are elicited [39]. Through control of cation influx, TRPM7 influences numerous cellular processes, including enzymatic activity, cytoskeletal organization, cell proliferation, migration, and survival [40,41]. Dysregulation of TRPM7 channel activity has been implicated in various pathological conditions [42,43,44].

### 2.3. TRPM7 Kinase Function

Each TRPM7 subunit has a C-terminal protein kinase domain with activity modulated by divalent cations: it is enhanced by Mg^2+^, inhibited by Zn^2+^, and is unaffected by Ca^2+^ [45]. TRPM7 kinase phosphorylates several protein substrates, including phospholipase C (PLC), nuclear histones, annexin I, and myosin IIA-C, that are involved in embryogenesis, lymphopoiesis, and cell adhesion and migration [8,9,33]. A screen of the rat library identified PLC as a partner interacting with the TRPM7 kinase [33]. Furthermore, TRPM7-kinase-regulated Ser/Thr phosphorylation in the C2 domain of PLCγ2 leads to reduced Ca^2+^ signaling in low Mg^2+^ conditions [46]. SMAD2 is a protein that plays a central role in the signaling pathway of transforming growth factor-beta (TGF-β) and is particularly important in driving the differentiation of T helper 17 (Th17) cells. An in vitro kinase assay using recombinant TRPM7 kinase, SMAD2, as well as C-terminal truncated SMAD2, revealed that TRPM7 phosphorylates normal SMAD2 but not the truncated SMAD2 in a dose-dependent manner [23]. Furthermore, the C-terminal Ser465/467-motif of SMAD2 was identified as a novel substrate for the TRPM7 kinase. The kinase can also phosphorylate myelin basic protein as well as histone H3 on serine and threonine residues [47]. Two major sites of autophosphorylation (Ser1511 and Ser1567) were identified in vitro by mass spectrometry, and these sites were found to be phosphorylated in intact cells [45]. The channel can autophosphorylate but autophosphorylation did not alter channel activity measured by whole-cell recording or Ca^2+^ influx [45].

### 2.4. TRPM7 Inhibitors and Activators

#### 2.4.1. TRPM7 Channel Inhibitors and Activators

TRPM7 channel inhibitors are structurally diverse and include both endogenous and exogenous molecules, with the majority being exogenous (see Table 1. compound structures acquired from PubChem compound database) [48]. Carvacrol (IC_50_ 306 µM), a natural monoterpenoid phenol, is a non-specific TRPM7 channel inhibitor [49]. Its suppression of TRPM7 causes cell cycle arrest at G0/G1 phases in cancer cells [50]. Another commonly used non-specific inhibitor is 2-APB (IC_50_ 70–170 µM) [34,51,52]. Its mechanism of action is acidifying the cytoplasm and consequently suppressing the TRPM7 channels [53]. SKF-96365, at 20 µM, has been reported to irreversibly inhibit the Mg^2+^-inhibited cation (MIC) current in basophilic leukemia cells [54,55]. The delayed onset of inhibition supports an indirect mechanism, rather than a direct interaction with the channel [54,55]. The biophysical properties of the MIC closely resemble those of cloned and heterologously expressed TRPM7 channels, suggesting that MIC current is mediated by TRPM7. However, more rigorous validation is required, including genetic approaches such as TRPM7 knockdown or knockout, as well as reconstitution experiments using heterologously expressed TRPM7 channels. Several 5-lipoxygenase inhibitors have been tested for their effects on TRPM7 in HEK-293 cells. Among them, NDGA, AA861, and MK886 potently inhibit TRPM7 channels [56]. In contrast, 5,6-dehydroarachidonic acid and zileuton are ineffective in suppressing TRPM7 activity. These data suggest that TRPM7 inhibition may not be directly linked to 5-lipoxygenase pathway blockade, but rather depends on the structural or pharmacological properties of individual inhibitors [56].

Waixenicin A, a natural marine product isolated from the soft coral *Sarcothelia edmondsoni*, is a potent inhibitor of both heterologously expressed and native TRPM7 channels [41]. Its inhibitory effect is strongly dependent on intracellular Mg^2+^, showing marked synergy with Mg^2+^ levels. Under physiological conditions (~700 μM Mg^2+^), waixenicin A inhibits TRPM7 with high potency (IC_50_ ≈ 16 nM), whereas removal of Mg^2+^ significantly reduces its efficacy (IC_50_ ≈ 7 μM) [41]. With physiological concentrations of intracellular Mg^2+^, waixenicin A (10 μM) completely abolished TRPM7 current; however, its inhibitory effect was reduced to ~50% upon intracellular Mg^2+^ depletion. This Mg^2+^-dependent modulation appears to involve the TRPM7 kinase domain, with Lys-1648 identified as a critical residue. Notably, waixenicin A exhibits relative selectivity for TRPM7 over TRPM6 and other TRP channels, making it one of the first relatively specific pharmacological inhibitors of TRPM7 [57]. Bioassays demonstrated that it inhibited cell proliferation by blocking the TRPM7 channel [41,57]. TRPM7 channel activity is also suppressed by endogenous regulators, including spermine and sphingosine, as well as by the immunosuppressant FTY720 (fingolimod), all of which exhibit IC_50_ values in the low micromolar range [58]. NS8593 is a TRPM7 channel antagonist that produces a full and reversible block of native TRPM7-like currents in HEK293 cells, smooth muscle cells, primary podocytes, and ventricular myocytes [59,60,61]. The inhibitory effect of NS8593 on TRPM7 is modulated by intracellular Mg^2+^ levels. Increasing intracellular Mg^2+^ (e.g., 300 μM) reduces its potency, resulting in a ~3.7-fold increase in IC_50_. In contrast, a pore-region mutation (Y1049P) enhances channel sensitivity to NS8593, producing an approximately fourfold decrease in IC_50_, indicating that the pore loop contributes to drug–channel interactions. Structure–activity relationship analysis further suggests that the benzimidazole moiety is critical for the pharmacological action of NS8593 on TRPM7 [59,60,61].

In contrast to TRPM7 channel inhibitors, small molecule compounds such as mibefradil, naltriben, and NNC55-0396 have been reported to activate the TRPM7 channels [62]. Interestingly, mibefradil and its homolog NNC 55–0396 share structural similarity with NS8593, a known inhibitor of TRPM7, suggesting that these compounds may interact with a common ligand-binding site but produce distinct functional effects on the channel activity [63]. Mibefradil enhances TRPM7 channel activity in a rapid, partially transient, and fully reversible manner. Its effect occurs at physiological concentrations of intracellular Mg^2+^ but is abolished by elevated Mg^2+^, indicating strong Mg^2+^ dependence [27]. By comparison, naltriben has been identified as the first drug-like activator of TRPM7 and can stimulate the channel activity even in the presence of high intracellular Mg^2+^ levels [62]. These findings support the existence of at least two classes of TRPM7 agonists: Mg^2+^-independent, exemplified by naltriben, and Mg^2+^-dependent, represented by mibefradil.

#### 2.4.2. TRPM7 Kinase Modulators

Pharmacological targeting of the TRPM7 kinase domain has been explored using high-throughput screening approaches. A kinase inhibitor library comprising 172 compounds was evaluated using an in vitro TRPM7 kinase assay, with rottlerin serving as a reference inhibitor. Compounds that reduced TRPM7 kinase activity to ≤70% at 10 μM were classified as hits, including TG100-115, TG100713, JNJ-07706621, PHA-665752, and butein [64]. Among these, TG100-115 emerged as the most potent inhibitor, with an IC_50_ of approximately 1.07 μM, markedly lower than that of rottlerin (IC_50_ ≈ 79 μM) [64,65]. Further docking analysis indicated that TG100-115 fits well within the ATP-binding pocket of the TRPM7 kinase domain. Consistent with this, increasing ATP concentrations resulted in a corresponding increase in IC_50_ values, indicating that TG100-115 acts as a competitive inhibitor at the ATP-binding site [65]. Notably, this study also demonstrated that TG100-115 inhibits TRPM7 channel activity, although the role of the TRPM7 kinase domain in regulating channel function remains controversial, with conflicting evidence from in vitro and in vivo studies suggesting that such effects may arise from direct or off-target actions. Collectively, these findings highlight TG100-115 as a relatively potent pharmacological tool for investigating TRPM7 kinase function, suggesting that it may serve as a lead compound for the development of more potent TRPM7 kinase inhibitors [64].

Despite the increasing availability of TRPM7 modulators, caution is needed when interpreting pharmacological studies because many compounds, including 2-APB, SKF-96365, and carvacrol, lack high selectivity and may affect multiple molecular targets. Even relatively selective agents such as waixenicin A, NS8593, and TG100-115 have important limitations, including Mg^2+^-dependent activity or potential off-target effects. Therefore, pharmacological findings should be complemented by genetic approaches, such as TRPM7 knockout, knockdown, or kinase-dead mutations, to more definitively establish TRPM7-dependent mechanisms.

**Table 1:** TRPM7 Modulators and Mechanisms of Action.

Modulatory Function	Compound Name	Structure	IC_50_/EC_50_ or Concentration Range	Mechanism of Action	Ref.
**Channel Inhibitors**					
	Carvacrol	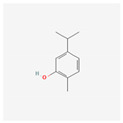	306 µM	Direct channel blockade	[49]
	2-APB	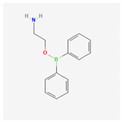	70–170 µM	Reversible inhibition through an intracellular acidification	[53]
	SKF-96365	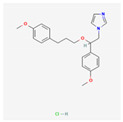	20 µM tested; IC_50_ not determined	Likely indirect channel blockade	[54,55]
	NDGA	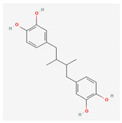	10 µM tested; IC_50_ not determined	Direct channel blockade	[44,56]
	AA861	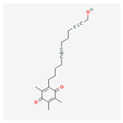	10 µM tested; IC_50_ not determined	Direct channel blockade	[44,56]
	MK886	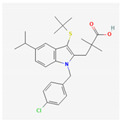	20 µM tested; IC_50_ not determined	Direct channel blockade	[44,56]
	Waixenicin A	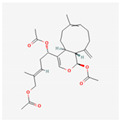	16 nM and 7.0 µM depending on intracellular Mg^2+^ levels	Mg^2+^-dependent reversible inhibition	[41]
	Spermine	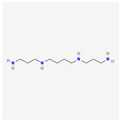	0.2–20 µM tested; IC_50_ not determined	Direct voltage-dependent channel blockade	[66]
	NS8593	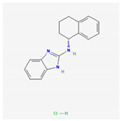	1.6 µM	Mg^2+^-dependent inhibition	[59,60,61]
	FTY720	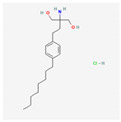	0.72 µM	Direct channel inhibition by reducing the open probability	[58]
	Sphingosine	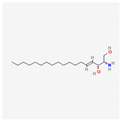	0.59 µM	Direct channel inhibition by reducing the open probability	[58]
**Channel Activators**					
	Mibefradil	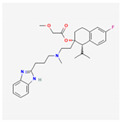	53 µM	Mg^2+^-dependent channel agonist	[63]
	Naltriben	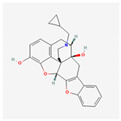	20.7 µM	Mg^2+^-independent, direct agonist	[62]
	NNC55-0396	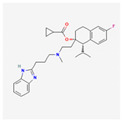	50 µM tested; EC_50_ not determined	Direct agonist	[63]
**Kinase Inhibitor**					
	Rottlerin	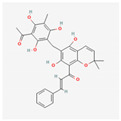	79 µM	Inhibits the phosphotransferase activity, may involve competition at ATP binding site	[64]
	NH125	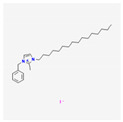	55 µM	Inhibits the phosphotransferase activity, likely functions as a non-specific colloidal aggregator	[67]
	TG100-115	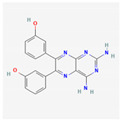	1.07 µM	ATP-competitive inhibitor	[64]
	TG100713	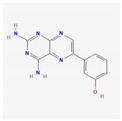	9.06 µM	ATP-competitive inhibitor	[64]
	JNJ-07706621	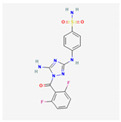	16.24 µM	ATP-competitive inhibitor	[64]
	PHA-665752	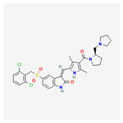	42.18 µM	ATP-competitive inhibitor	[64]
	Butein	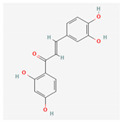	71.28 µM	ATP-competitive inhibitor	[64]

## 3. Role of TRPM7 in Immune Cells

### 3.1. TRPM7 Modulates Neutrophil Function

Neutrophils are polymorphonuclear leukocytes derived from myeloid progenitors found in the bone marrow [68]. They are constantly produced by bone marrow and account for up to 50–70% of all circulating leukocytes. Neutrophils are an important component of the innate immunity and respond rapidly to inflammatory signals and pathogens, contributing to the clearance of acute inflammation and bacterial infection; however, excessive neutrophil infiltration accelerates tissue damage due to unrestrained inflammation in pro-inflammatory as well as autoimmune diseases [68]. Compelling evidence has highlighted that Ca^2+^-signaling governs the key processes associated with neutrophil functions, such as NADPH oxidase activation and phagocytosis [69], suggesting that targeting Ca^2+^ influx in neutrophils has therapeutic potential for inflammatory diseases associated with excessive neutrophil activation. A precise understanding of calcium signaling mechanisms in neutrophils is therefore needed. In addition to Mg^2+^ and Zn^2+^, the TRPM7 channel is permeable to Ca^2+^ [33]. A TRPM7-mediated Ca^2+^ signal has been shown to regulate the migration of several cell types [70,71]. In neutrophils isolated from patients with rheumatoid arthritis (RA), TRPM7 was observed to mediate the CD147-driven enhancement of calcium-induced chemotaxis, adhesion, and invasiveness [72]. Another study showed the protective effects of salvianolic acid B on sepsis-induced acute lung injury through decreasing the expressions of TRPM7, probably due to a downregulation of TRPM7-mediated neutrophil migration and infiltration [73].

In addition to the TRPM7 channel-mediated Ca^2+^ signaling, TRPM7 kinase has also been suggested to contribute to neutrophil activation. When TRPM7 channel activity is inhibited by NS8593 or its kinase activity by TG100-115, human neutrophils are unable to transmigrate along a CXCL8 chemokine gradient or generate reactive oxygen species in response to Gram-negative bacterial lipopolysaccharide (LPS) [65]. TRPM7 kinase-deficient murine neutrophils also displayed a similar phenotype [65]. TG100-115 effectively reduced LPS-induced neutrophil chemotaxis as well as ROS production, most likely due to an inhibition of TRPM7-kinase/Akt/mTOR signaling pathways [65]. These studies highlight that TRPM7 may represent a potential target for treating unwanted excessive neutrophil activation.

### 3.2. TRPM7 Modulates Lymphocyte Function

Lymphocytes originate from bone marrow and differentiate from hematopoietic stem cells. Broadly, lymphocytes are categorized into B cells, T cells, and natural killer (NK) cells. They play a crucial role in the adaptive immune system, responsible for recognizing, responding to, and remembering specific antigens [74].

Evidence suggests that TRPM7 is fundamental to B cell biology, including cell development, antigen internalization, and presentation. Deletion of TRPM7, for example, caused Mg^2+^ deficiency, growth arrest, and cell death in DT40 B-lymphocytes within 24–48 h [75]. Animal studies also showed that expression of TRPM7 was required for B-cell development in vivo [21]. Supplementation with a high concentration of extracellular Mg^2+^ partially rescued the development of TRPM7-deficient B-cells in vitro [21]. The loss of TRPM7 kinase activity alone did not affect the development of B cells in the bone marrow. These findings highlight the TRPM7 channel’s indispensable role in B-cell development, primarily by maintaining intracellular Mg^2+^ homeostasis required for proliferation and survival. TRPM7 has also been demonstrated to play a role in B-cell antigen uptake and presentation [22]. DT40 B cells deficient in TRPM7 channel or kinase activity showed defective gathering of antigen [22]. Blocking TRPM7 function also compromised antigen internalization and presentation to T-cells [22]. These findings suggest that TRPM7 plays an important role in the processes required for B-cell maturation and the production of high-affinity antibodies.

In addition to B-cells, the TRPM7 channel-kinase is also crucial for T-cell function. Deletion of *Trpm7* in the T-cell lineage resulted in impairment of T-cell development in the thymus and altered chemokine and cytokine expression profiles [76]. In mice with kinase-dead *Trpm7^R/R^* (K1646R point mutation), the number of T-cells within intra-epithelial lymphocytes (IELs) and lamina propria lymphocytes (LPLs) was decreased, with CD4^+^ T-cells the most significantly affected compared to WT mice. Furthermore, in the few CD4^+^ cells recovered from the gut of *Trpm7^R/R^* mice, the analysis of functional subsets revealed a dramatic reduction of T_H_17 cells, indicating that TRPM7 kinase activity is essential for gut colonization by T cells and T_H_17 cell differentiation [23]. TGF-β/SMAD pathways are crucial for the polarization of CD4^+^ T-cells into T_H_17 cells [77]. A reduction in SMAD2 (Ser465/467) phosphorylation, which is essential for its transcriptional activity, was revealed in TGF-β1-treated *Trpm7*^R/R^ naive CD4^+^ T cells [77]. It has been shown that TRPM7 kinase can modulate SMAD2 signaling via direct phosphorylation at the C-terminal Ser465/467 motif. These findings suggest that the TRPM7 kinase regulates T_H_17 cell differentiation via TGF-β/SMAD2-dependent pathways [23].

### 3.3. TRPM7 Modulates Macrophage Function

Macrophages are a highly diverse group of plastic leukocytes. They participate in various immune responses, mounting distinct M1 or M2 phenotypes to maintain homeostasis. Stimulation with pro-inflammatory particles, LPS, or IFN-γ, promotes polarization toward the M1 pro-inflammatory phenotype, while stimulation with interleukin-4 (IL-4) drives polarization toward the anti-inflammatory M2 phenotype [78]. Emerging evidence indicates that TRPM7 plays a critical role in regulating macrophage function. TRPM7 expression is preferentially elevated in M1 macrophages compared with M2 macrophages, suggesting its association with pro-inflammatory activation [78]. Pharmacological blockade of TRPM7 shifts macrophage polarization toward an anti-inflammatory phenotype, characterized by reduced expression of M1 markers, including TNF-α and nitric oxide synthase (iNOS), and increased expression of M2 markers such as arginase-1 (Arg-1) and CD206 [78]. Consistent with this role, another study reported that TRPM7-deficient macrophages exhibit significantly reduced LPS-induced inflammatory gene expression and production of key pro-inflammatory cytokines such as IL-1β [79]. TRPM7-mediated Ca^2+^ influx is required for TLR4 endocytosis and downstream IRF3 and NF-κB activation, and mice with myeloid-specific deletion of Trpm7 are protected from LPS-induced peritonitis [79]. Collectively, these findings identify TRPM7 as a key regulator of macrophage activation/polarization and highlight its potential as a therapeutic target for inflammatory diseases [78].

### 3.4. TRPM7 Modulates Microglia Function

In contrast to the peripheral immune cell populations, the functions of TRPM7 in the CNS immune cells, particularly microglia, remain comparatively underexplored. Microglia comprise approximately 5–15% of all brain cells and serve as the resident innate immune sentinels of the CNS [80]. Through continuous surveillance of the neural microenvironment, they balance host defense and maintain tissue homeostasis. When dysregulated, microglia become potent drivers of neuroinflammation, contributing to neurodegeneration in both acute neurological conditions such as stroke and traumatic brain injury, and chronic disorders such as Alzheimer’s disease, PD, and amyotrophic lateral sclerosis [80,81,82,83]. Modulating microglia reactive states is an attractive therapeutic strategy to mitigate neuroinflammation and slow disease progression. TRPM7 transcripts and TRPM7-like currents were originally identified in the rat microglia cells [84]. The recorded currents display hallmark biophysical and pharmacological features of TRPM7 currents, including strong outward rectification above +50 mV, inhibition by intracellular Mg^2+^, and sensitivity to the non-specific TRPM7 inhibitor 2-APB [53]. Given the channels’ permeability to Ca^2+^, which is crucial for microglia function, TRPM7-mediated calcium entry may contribute to microglia functions that rely on intracellular Ca^2+^ signaling [85]. The newly identified TRPM7 channel inhibitor VPC01091.4 (VPC), which can effectively accumulate in the brain, exerts robust anti-inflammatory effects on microglia by reducing LPS-induced expression of IL-1β and IL-6 in the brain [86]. Our own studies demonstrate that the TRPM7-selective channel inhibitor waixenicin A and TRPM7-kinase inhibitor TG100-115 significantly reduce LPS-stimulated migration, phagocytosis, and IL-6 secretion in human HMC3 microglia [87]. These pharmacological studies support a potential role for TRPM7 in regulating microglial inflammatory responses.

### 3.5. Role of TRPM7 in Astrocytes

Astrocytes or astroglia are star-shaped glia in the CNS, ensheathing synapses and blood vessels, where they maintain ionic, metabolic, and neurotransmitter homeostasis. In response to inflammatory cues, they switch to context-dependent reactive states that can support tissue protection and repair; however, when dysregulated, these states can amplify proinflammatory signaling, disrupting CNS homeostasis [88]. In reactive astrocytes within multiple sclerosis (MS) lesions and in primary astrocytes under chronic inflammatory conditions, TRPM7 expression was markedly enriched [58,89]. Overexpression of TRPM7 in astrocytes impaired neuronal outgrowth in vitro by increasing the production of chondroitin sulfate proteoglycans, a major structural component of the gliotic scar [89]. These findings indicate that astrocytic TRPM7 is a critical regulator of glia scar formation, revealing a novel mechanism by which reactive astrocytes affect neuronal outgrowth. Furthermore, TRPM7 was markedly upregulated in astrocytes after spinal cord injury; inhibiting TRPM7 downregulated the production of TNF-α, IL-6, IL-1β, and matrix metalloproteinase 9 (MMP9), which are known inflammatory factors involved in the induction and/or maintenance of neuropathic pain after injury [90].

## 4. Role of TRPM7 in Inflammatory Modulation in Neurological Diseases

Epidemiologic studies have performed comparative gene expression analyses that link TRPM7 to brain-related diseases such as multiple sclerosis, Alzheimer’s disease, and stroke [91]. Based on the emerging evidence of TRPM7 in microglia and astrocytes as discussed above [86,90], dysregulated TRPM7 activity may contribute to neuroinflammatory processes associated with neurodegeneration. However, much of the current evidence is derived from pharmacological studies, and future investigations using cell-specific genetic models will be required to establish the causal role of TRPM7 in disease pathogenesis. Microglia during neurological disorders have been shown to experience excessive reactivity [92]. Activated microglia release a range of cytokines, including TNF-α, IL-1β, and IL-6, that dynamically regulate neuroinflammatory responses [80,93,94]. In addition, they also release MMP9, which can cause the degradation of the BBB [93,95]. BBB disruption is observed in both acute brain injuries (e.g., stroke and traumatic brain injury) and neurodegenerative diseases (e.g., AD and MS). It permits peripheral immune cell infiltration into the brain, further amplifying inflammatory responses and contributing to secondary neuronal injury/degeneration.

Neuroimmune interactions involve complex communication among microglia, astrocytes, neurons, and infiltrating peripheral immune cells that collectively regulate inflammatory responses within the CNS. Activated microglia release pro-inflammatory cytokines and chemokines through signaling pathways such as NF-κB, MAPK, and NLRP3 inflammasome activation, which promote astrocytic activation and inflammatory reactivity [82,96]. Astrocytes further modulate neuroimmune responses through JAK/STAT3-mediated signaling [88]. Dysregulation of these microglial and astrocytic signaling pathways has been implicated in persistent neuroinflammation, BBB disruption, and progressive neuronal injury in both acute and chronic neurological disorders [94,96].

For example, the interactions between CNS-resident astrocytes and microglia, and infiltrating T cells, have been linked to the persistence of inflammation and impaired tissue repair in ischemic stroke [97]. These CNS-infiltrating T cells express activation markers (e.g., CD44 and CD25) and produce high levels of inflammatory cytokines, including IFN-γ, IL-17, and TNF-α [97]. These cytokines will interact with astrocytes and microglia, leading to their pro-inflammatory activation, prolonging injury, and preventing anti-inflammatory reactivity. It should also be noted that ischemic stroke induces the long-term activation of T cells that persists for years after injury, leading to continued inflammation in the CNS [97]. Similarly, neutrophils and B cells also migrate and invade the brain in both acute and chronic CNS diseases, and have been shown to contribute to CNS inflammation and neurodegeneration [98]. Considering that both the TRPM7 channel and the kinase are implicated in the immune responses, a combination of inhibitors for both the TRPM7 channel and the kinase may obtain a maximal anti-inflammatory activity. However, considering that TRPM7 also plays a critical physiological role in neuronal activity, such as synaptic transmission and astrocyte physiology [62,89,99,100], the allosteric modulators that can suppress the over-activation of TRPM7, but keep a basal activity, may help reduce the potential side effects.

## 5. Conclusions and Perspectives

This review provides an overview of the current understanding of TRPM7 structure, function, and pharmacology, with particular emphasis on its emerging roles in both peripheral and central immune cells. Compared with TRPM7’s role in peripheral immune cells, its function in central immune cells is poorly understood, particularly under various CNS disease conditions. Accumulating evidence implicates TRPM7 in the pathophysiology of several neurological diseases, including ischemic brain injury, ALS, PD, and AD, although the underlying mechanisms remain incompletely understood [101,102,103]. Previous studies focused on the neuroprotective activity of the TRPM7 channel blockade, e.g., via reducing the Ca^2+^ and Zn^2+^ toxicity [17,18]. However, this neuron-centric view may be incomplete, as TRPM7 also regulates signaling pathways in immune cells, as well as cytokine production and inflammatory activation. As a chanzyme, TRPM7 is uniquely positioned at the intersection of ion homeostasis and intracellular signaling, potentially linking ionic dysregulation to immune responses in the central nervous system. Future studies focusing on cell-type-specific functions, as well as distinguishing between its channel and kinase activities, will be critical for advancing our understanding of TRPM7 biology and its therapeutic potential.

## Data Availability

No new data were created or analyzed in this study. Data sharing is not applicable to this article.

## Abbreviations

The following abbreviations are used in this manuscript:

ChaK1	Channel-Kinase 1
LTRPC7	Long Transient Receptor Potential Channel 7
MHR	Melastatin Homology Region
TRP-PLIK	Transient Receptor Potential-Phospholipase C-Linked Kinase
